# Supplementary material for: Differences in cervical sagittal parameters and muscular function among subjects with different cervical spine alignments: a surface electromyography-based cross-sectional study
Source: PeerJ. 2024 Sep 25;12:e18107. doi: 10.7717/peerj.18107 (PMC11438432; doi:10.7717/peerj.18107)
Supplement: Supplemental Information 2 [file peerj-12-18107-s002.doc]

STROBE Statement—Checklist of items that should be included in reports of ***cross-sectional studies***

|  | Item No | Recommendation | where in the manuscript each item is addressed |
| --- | --- | --- | --- |
| **Title and abstract** | 1 | (*a*) Indicate the study’s design with a commonly used term in the title or the abstract | ☑Title |
|  |  | (*b*) Provide in the abstract an informative and balanced summary of what was done and what was found | ☑ |
| Introduction | | |  |
| Background/rationale | 2 | Explain the scientific background and rationale for the investigation being reported | ☑ P4-5 line 46-78 |
| Objectives | 3 | State specific objectives, including any prespecified hypotheses | ☑ P5 line 78-88 |
| Methods | | |  |
| Study design | 4 | Present key elements of study design early in the paper | ☑ P8-10 line 146-180 |
| Setting | 5 | Describe the setting, locations, and relevant dates, including periods of recruitment, exposure, follow-up, and data collection | ☑P10-11 line 193-206 |
| Participants | 6 | (*a*) Give the eligibility criteria, and the sources and methods of selection of participants | ☑P6 line 92-96  P10 line 194 |
| Variables | 7 | Clearly define all outcomes, exposures, predictors, potential confounders, and effect modifiers. Give diagnostic criteria, if applicable | ☑P8 line 131-144 |
| Data sources/ measurement | 8* | For each variable of interest, give sources of data and details of methods of assessment (measurement). Describe comparability of assessment methods if there is more than one group | ☑ P8-10 line 146-180 |
| Bias | 9 | Describe any efforts to address potential sources of bias | ☑P10-11 line 193-206 |
| Study size | 10 | Explain how the study size was arrived at | ☑P7 line 120-128 |
| Quantitative variables | 11 | Explain how quantitative variables were handled in the analyses. If applicable, describe which groupings were chosen and why | ☑P10 line 182-189 |
| Statistical methods | 12 | (*a*) Describe all statistical methods, including those used to control for confounding | ☑P10 line 182-189 |
|  |  | (*b*) Describe any methods used to examine subgroups and interactions | ☑P10 line 182-189 |
|  |  | (*c*) Explain how missing data were addressed | ☑P6 line 101-103 |
|  |  | (*d*) If applicable, describe analytical methods taking account of sampling strategy | ☒consecutively enrolled |
|  |  | (*e*) Describe any sensitivity analyses | ☒none |
| Results | | |  |
| Participants | 13* | (a) Report numbers of individuals at each stage of study—eg numbers potentially eligible, examined for eligibility, confirmed eligible, included in the study, completing follow-up, and analysed | ☒consecutively enrolled cross-sectional study |
|  |  | (b) Give reasons for non-participation at each stage | ☒consecutively enrolled cross-sectional study |
|  |  | (c) Consider use of a flow diagram | ☑ Figure 2 |
| Descriptive data | 14* | (a) Give characteristics of study participants (eg demographic, clinical, social) and information on exposures and potential confounders | ☑P10-11 line 193-206 |
|  |  | (b) Indicate number of participants with missing data for each variable of interest | ☑P10-11 line 193-206 |
| Outcome data | 15* | Report numbers of outcome events or summary measures | ☑P10-11 line 193-206 |
| Main results | 16 | (*a*) Give unadjusted estimates and, if applicable, confounder-adjusted estimates and their precision (eg, 95% confidence interval). Make clear which confounders were adjusted for and why they were included | ☑P11-14 |
|  |  | (*b*) Report category boundaries when continuous variables were categorized | ☒ None variables were categorized |
|  |  | (*c*) If relevant, consider translating estimates of relative risk into absolute risk for a meaningful time period | ☒ not inculded in our study |
| Other analyses | 17 | Report other analyses done—eg analyses of subgroups and interactions, and sensitivity analyses | ☑P14-15 |
| Discussion | | |  |
| Key results | 18 | Summarise key results with reference to study objectives | ☑P16-20 line 316-396 |
| Limitations | 19 | Discuss limitations of the study, taking into account sources of potential bias or imprecision. Discuss both direction and magnitude of any potential bias | ☑P20 line 397-402 |
| Interpretation | 20 | Give a cautious overall interpretation of results considering objectives, limitations, multiplicity of analyses, results from similar studies, and other relevant evidence | ☑P20 line 404-414 |
| Generalisability | 21 | Discuss the generalisability (external validity) of the study results | ☑P15-16 line 292-315 |
| Other information | | |  |
| Funding | 22 | Give the source of funding and the role of the funders for the present study and, if applicable, for the original study on which the present article is based | ☑P21 line 419-421 |

*Give information separately for exposed and unexposed groups.

**Note:** An Explanation and Elaboration article discusses each checklist item and gives methodological background and published examples of transparent reporting. The STROBE checklist is best used in conjunction with this article (freely available on the Web sites of PLoS Medicine at http://www.plosmedicine.org/, Annals of Internal Medicine at http://www.annals.org/, and Epidemiology at http://www.epidem.com/). Information on the STROBE Initiative is available at www.strobe-statement.org.
